# Non-Invasive Continuous Glucose Monitoring with Multi-Sensor Systems: A Monte Carlo-Based Methodology for Assessing Calibration Robustness

**DOI:** 10.3390/s130607279

**Published:** 2013-06-03

**Authors:** Mattia Zanon, Giovanni Sparacino, Andrea Facchinetti, Mark S. Talary, Martin Mueller, Andreas Caduff, Claudio Cobelli

**Affiliations:** 1 Department of Information Engineering, University of Padova, via Gradenigo 6B, Padova 35131, Italy; E-Mails: zanonma1@dei.unipd.it (M.Z.); gianni@dei.unipd.it (G.S.); facchine@dei.unipd.it (A.F.); 2 Biovotion AG, Technoparkstrasse 1, Zurich 8005, Switzerland; E-Mails: mark.talary@biovotion.com (M.S.T.); martin.mueller@biovotion.com (M.M.); andreas.caduff@biovotion.com (A.C.)

**Keywords:** diabetes, model, multisensor

## Abstract

In diabetes research, non-invasive continuous glucose monitoring (NI-CGM) devices represent a new and appealing frontier. In the last years, some multi-sensor devices for NI-CGM have been proposed, which exploit several sensors measuring phenomena of different nature, not only for measuring glucose related signals, but also signals reflecting some possible perturbing processes (temperature, blood perfusion). Estimation of glucose levels is then obtained combining these signals through a mathematical model which requires an initial calibration step exploiting one reference blood glucose (RBG) sample. Even if promising results have been obtained, especially in hospitalized volunteers, at present the temporal accuracy of NI-CGM sensors may suffer because of environmental and physiological interferences. The aim of this work is to develop a general methodology, based on Monte Carlo (MC) simulation, to assess the robustness of the calibration step used by NI-CGM devices against these disturbances. The proposed methodology is illustrated considering two examples: the first concerns the possible detrimental influence of sweat events, while the second deals with calibration scheduling. For implementing both examples, 45 datasets collected by the Solianis Multisensor system are considered. In the first example, the MC methodology suggests that no further calibration adjustments are needed after the occurrence of sweat events, because the “Multisensor+model” system is able to deal with the disturbance. The second case study shows how to identify the best time interval to update the model's calibration for improving the accuracy of the estimated glucose. The methodology proposed in this work is of general applicability and can be helpful in making those incremental steps in NI-CGM devices development needed to further improve their performance.

## 1. Introduction

Diabetes consists in a chronic malfunction of the glucose-insulin regulatory system leading to the onset of long and short term health threats caused by uncontrolled excursions of blood glycaemic levels outside the normal “euglycaemic” range (70 ÷ 180 mg/dL) [1]. In the last few decades, diabetes has become a major disease in the rich countries and received an increasing amount of attention because of both its social and economic implications, with more than 439 million of cases estimated in 2030 [2]. Glucose sensors can play a crucial role for improving diabetes treatment. In particular, continuous glucose monitoring (CGM) sensors have been on the market since the early 2000s and are of great interest for several reasons related to the retrospective tuning and optimization of diabetes therapy, as well as for on-line applications such as the so called “artificial pancreas” or hypo/hyper glycemic event prediction [3–10]. Most of the CGM sensors exploit an enzyme based glucose-oxidase needle electrode and are thus invasive, although minimally. To overcome the invasiveness of the needle-based methods, in the last decade several non-invasive continuous glucose monitoring (NI-CGM) technologies have been also proposed (see [11] for a recent overview).

NI-CGM sensors exploit different measurement techniques such as optical, electromagnetic, electrical as well as acoustic and thermal (see [12,13] for reviews). NI-CGM is appealing for obvious reasons related to patient comfort, although current accuracy is not yet comparable with that of enzyme-based needle sensors which measure in the subcutis. However they achieved good accuracy in glucose trend estimation [14], making it a valid complement to standard fingerprick devices that could greatly help the diabetic patient in preventing the occurrence of critical events, such as hypoglycaemia.

One major difficulty met in developing NI-CGM sensors consists in dealing with some environmental and physiological processes, e.g., blood perfusion, temperature variations and sweating (a very common source of disturbance in daily-life conditions) that act as perturbing factors. These non-glucose related processes restrict the domain of applicability of NI-CGM sensors, making their accuracy acceptable only during in-clinic studies [15,16]. For this reason, a recently proposed approach for NI-CGM aims at combining different sensors within the same device in order to detect and compensate those disturbances responsible for the decreased accuracy [17–19].

To allow glucose to be estimated, measurements obtained from these multi-sensor devices need to be properly combined through suitable mathematical models. Usually, black-box models are considered, since quantitative physical descriptions of how the quantities non-invasively measured by the multisensor are linked to glucose are not available or have not yet shown significant advantages. Once the structure of this black-box model is fixed, e.g., with a multivariate linear regression model, several techniques such as partial least squares (PLS) or the least absolute shrinkage and selection operator (LASSO) can be considered for the estimation of model parameters from a suitable set of data. In order to be usable at an individual level, the combination “Multisensor + model” is calibrated by exploiting one or more reference blood glucose (RBG) values measured by the patient with standard enzyme-based fingerprick devices [17–20].

The robustness of the aforementioned calibration procedure is crucial for the potential practical use of NI-CGM multi-sensor devices. To the best of our knowledge, no methodologies have been proposed so far in the literature to assess the goodness of the calibration schemes of these devices and trial-and-error procedures represent the routine. This work presents a Monte Carlo (MC) methodology for such an application. MC techniques have been applied to other problems in the context of CGM sensors for diabetes management, e.g., [21–24], but never to assess calibration robustness. Two specific case studies will be discussed in the present work. In the first, we will assess the robustness of calibration against perturbing processes (e.g., sweat events) that could deteriorate accuracy of estimated glucose profiles. In the second, we will use the MC simulation to evaluate alternative strategies (e.g., repeated calibration) usable for calibrating NI-CGM devices. Both examples will exploit data recorded from the multi-sensor system developed by Solianis Monitoring AG (Zurich, Switzerland) and now owned by Biovotion AG (Zurich, Switzerland). While the present work does not develop any new MC-based mathematical methodology, it demonstrates how a well-established technique can be usefully employed in the investigation of a key issue in the development of multi-sensors for NI-CGM (specifically related to calibration requirements). The MC based technique considered in this work leads to a better quantitative understanding of the strengths and drawbacks of NI-CGM technologies and helps their development by evidencing possible margins of improvement.

## 2. Case Studies and Problem Statement

The strategy devised in the present work to assess the calibration of multi-sensor devices for NI-CGM is of general usability, but it is convenient to present it by making reference to a specific system (see Section 2.1). In particular, the Solianis multisensor NI-CGM device documented in [17], complemented by a model identified as recently discussed in [14] is considered. For the sake of brevity, in the following such a system will be referred to as “Multisensor” (with capital M). By considering data already published in [20], we will address: (a) how to assess the robustness of the calibration against sweat events (Section 2.2) and (b) how to determine if repeated calibration are needed and at which time instants for improving accuracy of estimated glucose (Section 2.3).

### 2.1. A Specific NI-CGM Multisensor Technology and its Calibration

#### 2.1.1. “Multisensor+model” Combination

**Figure 1.**
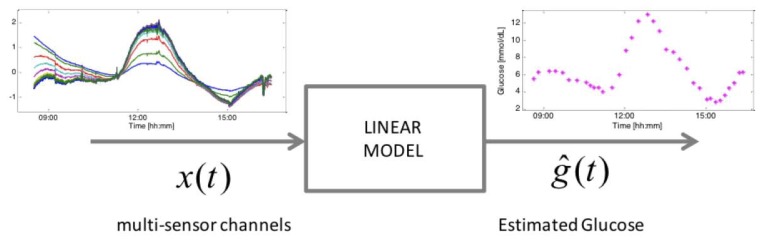
Example of multi-sensor data (**left**) that are combined through a proper mathematical model, in this case a multivariate linear regression model (**middle**), for estimating glucose profiles (**right**).

The considered device consists of several sensors embedded within the same device substrate for the bio-physical characterization of skin and underlying tissue in order to track glucose-related and perturbing processes. Dielectric spectroscopy (DS) electrodes of different shape and geometries, spanning different frequency ranges are included, as well as optical modules, temperature, humidity sensor and an accelerometer. These sensors allow the measurement of endogenous (skin perfusion, sweating, movement, *etc.*) as well as exogenous (temperature, humidity, *etc.*) factors that can influence the main glucose related signals [25]. The 150 channels measured by the Multisensor (Figure 1, left) allow glucose concentrations (Figure 1, right) to be inferred through a static black-box multivariate linear regression model (Figure 1, middle) (with order and parameters common to the entire population of subjects) with parameter vector *β* in (1) identifiable by several approaches, among which the Least Absolute Shrinkage and Selection Operator (LASSO) is the one showing the best performance [14]. According to this model, the estimated glucose concentration at time t, *ĝ*(*t*), is given by:

(1) $$\hat{g}(t)=\beta x(t)+b$$

where ***x****(t*) is the vector collecting the 150 channel samples measured by the Multisensor at each time instant *t* and *b* is the baseline glucose calibration parameter calculated exploiting a single RBG provided by a “gold standard” technique requiring a blood sample obtained through a lancet pricking the skin. In particular, the calibration strategy taken into consideration involves an adjustment of the glucose baseline for the estimated glucose profile, namely, the glucose profile is shifted to the first value available by a quantity given by:

(2) b=βx(t)−g(t)

where *b* is the glucose baseline, calculated as the difference between the estimated glucose value given by the multivariate linear model *βx*(*t*) and the RBG point at the same time instant *g*(*t*). This initial adjustment is usually performed 75 min after the Multisensor is placed in contact with the skin. This time is required for allowing adaptation processes related to Multisensor-skin contact to complete [14]. This value is then kept fixed for all the time the Multisensor is worn.

*Ad hoc* investigations (outside the scope of the present work) could assess the potentiality of more sophisticated calibration approaches, e.g., [26–28]. However, the simple calibration rule, incorporated in (1), and suggested by the manufacturers of the Multisensor, is used in this paper without loss of generality. Indeed, the MC methodology we will describe can deal with any calibration law.

#### 2.1.2. Database and Study Design

The database, taken from [20], consists of data from 45 experimental datasets during which Multisensor data, collected at quasi continuous time (sampling rate 20 s), and RBG values, collected by finger prick with 10 to 20 min sampling rate, were acquired in parallel for (on average) eight hours in six diabetic subjects affected by type 1 diabetes mellitus whose plasma glucose was induced to vary according to a pre-determined profile. Glucose levels were induced to vary after 75 min to allow euglycaemic level to be established and Multisensor-skin contact processes to complete. For further details about the protocols and the data pre-processing we refer the reader to [20].

To identify and test models, the dataset is split into two parts (dataset part 1 and dataset part 2) by randomly pooling together different sessions, paying attention to have an approximately equal number of recordings per subject in each data subset. If the first part of the dataset (dataset part 1) is used for the identification of the multivariate linear regression model parameters, the second part (dataset part 2) is used to test the model over data not used during the model derivation stage and *vice versa*, the goal of this strategy being to perform a fully prospective analysis. Swapping the two datasets has the rationale of allowing us to verify the consistency of the results. Notably, the popular leave-one-out cross-validation strategy could not be considered here because the MC methodology would have been performed over a single experiment (the one left out during the cross-validation routine) that changes from iteration to iteration.

### 2.2. Influence of Sweat Events on Multisensor Performance

**Figure 2.**
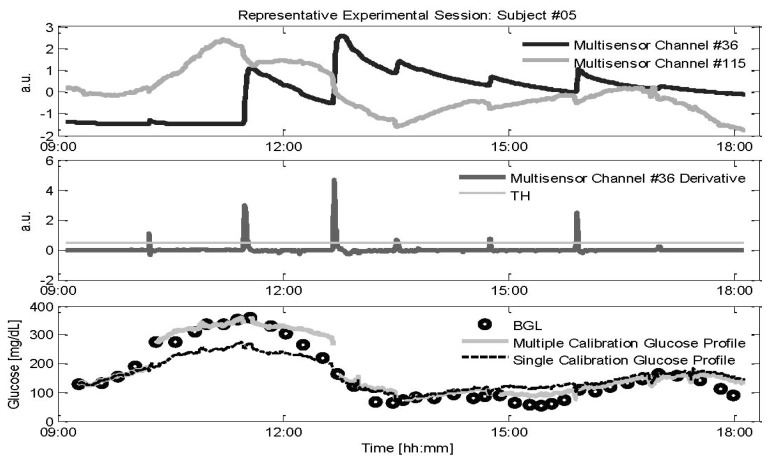
Representative experimental session of subject #05 where the estimated glucose profile is recalibrated after each detected sweat event. Top: Two of the 150 Multisensor channels recorded: channel #36 (black line) is sensitive to sweat events, and channel #115 (grey line) is particularly sensitive to glucose changes. Middle: derivative of the channel 36 signal (black line) with the chosen threshold TH (thin grey line). Bottom: Glucose profiles estimated by using single baseline calibration (black dashed line) and multiple calibrations (grey line). RBG samples collected in parallel are also shown to allow qualitative visual assessment of accuracy (black circles).

The parameter *b* in Equation (1) is estimated by the calibration procedure of Equation (2) at the beginning of each experimental session and is not updated for the entire duration of the recording, (*i.e.*, whilst the multi-sensor device remains in contact with the skin). However, uncontrollable events may occasionally disturb the multi-sensor monitoring. In particular, a sweat event involves the creation of a conductive saline layer at the sensor-skin interface. As long as the sweat activity diminishes, the signal is expected to return to a level close to its initial value. However, as shown in Figure 2 (top), there still could be a large off-set in the channels measuring sweats (interdigitated electrode with specific geometrical shape and exploiting specific frequency range, namely 1–200 KHz, for being sensitive to sweat, from now on identified as channel #36, black line) that, after the occurrence of a sweat event, does not always return to the value before the event (see just before and after 12:00 in Figure 2), a condition already observed in the literature [29]. This off-set, together with changes in the hydration levels of the skin and underlying tissues resulting from sweat, could also affect the DS electrodes measuring the main glucose related signals (see Figure 1 top, channel #115, grey line) despite the fact that these electrodes are designed to sample the most microvascularized area (*i.e.*, the upper and deep vascular plexus). The multivariate linear regression model used by the Multisensor is expected to properly combine the information contained in the multi-sensor channels to compensate non-glucose related physiological processes such as sweat events. However, the compensation of the effects of sweat events on the main glucose related signals that is expected to occur on the multi-sensor channels #36 (which contains information about the electrolyte balance changes on the skin surface) is principally performed by channels exploiting frequencies in the GHz range, that measure water balance variations in the tissue because sweating also results in changes in hydration. Assuming that the model is not able to properly compensate these sweat related processes, a new calibration point would be needed for re-adjusting the glucose baseline every time a sweat event is occurring. This need requires the collection of a new RBG sample obtained by blood fingerprick with some discomfort for the patient which reduces, in a practical perspective, the usefulness of NI-CGM.

### 2.3. Influence of Calibration Scheduling on Multisensor Performance

In general, it is well known from the literature how calibration parameters can vary over time due to several different reasons, for example, a degradation of the glucose enzyme in minimally-invasive devices [30], or for the effect of environmental and physiological processes that could make the parameters calculated when the device was worn no longer suitable to estimate accurate glucose levels in NI-CGM devices. Assessing the benefit of performing a scheduled re-calculation of the calibration parameter *b* can thus be useful in practice to evaluate the robustness of a NI-CGM multi-sensor.

## 3. Monte Carlo Methodology to Assess Effectiveness of Calibration Strategies

In this section we present how the MC simulation can be used to validate the usefulness of designed calibration of multi-sensor systems for NI-CGM to improve their accuracy. First, the two calibration strategies will be presented. Then, the MC simulation will be illustrated in detail.

### 3.1. Calibration after Sweat Events

As described in Section 2.2, if the effects of sweat events impair the calibration parameter calculated at the beginning of each experimental session, glucose levels after the occurrence of sweats could be estimated with less accuracy. Thus, we assess the possible benefits obtained by recalculating *b* in Equation (2) exploiting the first RBG samples collected after the occurrence of sweat events. The first problem is to identify a sweat event using the multi-sensor data that appear more sensitive to sweat. As shown in Figure 2, calculating the derivative (middle panel) of channel #36 (black line in top panel), provides an easy but effective procedure for the on-line detection of sweat events by setting a proper threshold (TH shown in grey in middle panel). Here the threshold is chosen, in a pool of candidate values, as the one giving the better trade-off between missed and identified sweat events. After a sweat event is detected, a new calculation of the calibration parameter is performed according to Equation (2): the new *b* is calculated at the time instant *t_i_* of the first available RBG after the detection of the sweat event.

In the following, the accuracy of glucose profiles in both configurations (single initial baseline calibration and multiple calibration after each sweat event) is measured through indexes widely used in the diabetes community, namely the Root Mean Squared Error $(\text{RMSE}=\sqrt{{\text{avg}}_{i}\;({\left(g_{i}-{\hat{g}}_{i}\right)}^{2}))}$, the Mean Absolute Differences (*MAD* = *avg_i_* (| *g_i_* − *ĝ_i_* |)) and the Mean Absolute Relative Differences (*MARD* = *avg_i_* (| *g_i_* − *ĝ_i_* | / *g_i_*)) where *g*(*t_i_*) and *ĝ*(*t_i_*) are respectively the RBG and NI-CGM values. These indexes give a measure of how close the estimated glucose profiles are to the RBG values, both in terms of relative and absolute values.

### 3.2. Multiple Calibration Scheduling

The second scenario evaluates the usefulness of performing a further calculation, after the initial one, of the calibration parameter, a strategy that is used for minimally-invasive CGM devices. In order to estimate the best value for *T_c_* for recalculating the calibration parameter *b*, we investigated a pool of possible candidates: *T_c_* = *1*,….,*7 h*. To find the best *T_c_*, the performance indexes are used to measure accuracy of the estimated glucose profiles that undergo the initial calibration followed by a recalculation of *b* according to *T_c_*. After the best time interval *T_c_* is found, a MC simulation is undertaken in order to verify whether the improvement was really due to the proposed calibration scheduling or rather to the consideration of more RBG points used for calibration. The quantification of improvement in accuracy is performed resorting to the same indexes introduced in Section 3.1.

### 3.3. The Monte Carlo Simulation

MC-based methods are a well-known and widely used class of techniques for solving different problems in engineering, physics, statistics and mathematical sciences, see e.g., [31,32]. For instance, MC methods are used to solve problems when an exact computation is not feasible, e.g., in numerical integration and numerical optimization. In statistics, a MC-based method is used to solve a permutation test which is a particular resampling technique for performing significance test analysis [33]. In general, MC methods consist of performing an elevated number of simulations (N) of an experiment, whose outputs are used to validate the goodness of the results obtained on the original experiment (e.g., by comparing the results on the basis of a selected outcome metric). The general formulation for a MC-based method follows some specific steps. A number N of simulations needs to be fixed (e.g., N = 1,000), together with the domain of the possible inputs (*i.e.*, the set of variables which vary from simulation to simulation). Then, for each simulation: (a) inputs are randomly drawn from a probability distribution over the domain; (b) the simulations are performed over the randomly chosen inputs, obtaining a set of outputs, on which a deterministic computation of the target outcome is performed. At the end of the N iterations, the distribution of the target outcome is derived aggregating the result of each iteration. To be more precise, in this work, the goal is to compare a single value, representing for example the mean accuracy obtained with a new calibration scheme over the test data set, with a distribution of mean accuracy values obtained during the MC simulations where glucose profiles are calibrated randomly. Thus, the MC technique is used to simulate the results that would be obtained by recalibrating randomly during the experimental sessions. These simulated results can then be used to validate the calibration scheme. In other words, the calibration scheme under analysis can be considered useful if there is a small percentage of MC iterations returning worse accuracy values than the calibration scheme under test.

**Figure 3.**
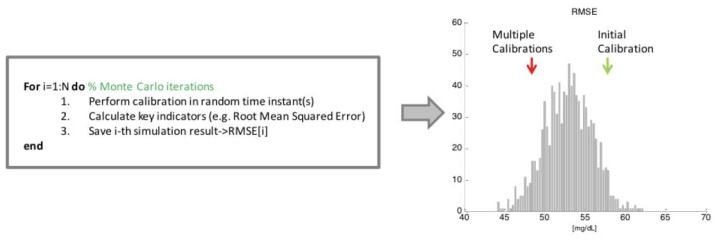
The box describes schematically the steps of the Monte Carlo (MC) methodology for assessing robustness of calibration. Each of the N iterations returns a measure of the accuracy for the glucose profiles calibrated in random time instant(s), RMSE in this case. The aggregate results of the MC simulation are then exploited to build the histogram of the sample distribution. The height of a grey bar represents the number of times that value of accuracy is obtained during the N iterations. The distribution is compared to the initial (green arrow) and multiple (red arrow) calibration results under test.

Figure 3 illustrates a scheme of the application of such methodology reported to our specific case study. In particular, the domain over which the inputs are sampled corresponds to the set of time instants *t_i_* where RBG values are available. At each iteration of the MC simulation (for loop of Figure 3), each glucose profile estimated by the multivariate model in the test data set undergoes the initial calibration (as explained in Section 2.1), which is fixed and does not change from iteration to iteration. Then, the calibration parameter *b* is recalculated, according to Equation (2), one or several times over a grid of random time instants, say N_s_, exploiting RBG values correspondent to time instants *t_i_* randomly drawn from the sample space. Note that the number of recalculations of the parameter *b* performed at each iteration, indicated with N_s_, is fixed and depends on the number of events that characterizes the scenario under analysis. In the sweat events scenario (see Section 3.1), *b* will be recalculated N_s_ times in random time instants within the experimental session, where N_s_ is the average number of sweat events occurring in the test data experimental sessions. On the other side, considering the multiple calibration scheduling scenario (Section 3.2), we will test the benefit of performing only one additional recalculation of *b* after a certain amount of time *T_c_* from the initial one, thus N_s_ = 1, meaning that for each MC iteration, every estimated glucose profile in the test data set undergoes only one additional recalculation of *b* in a random time instant. Accuracy of each glucose profile, obtained as the result of the deterministic calculation exploiting the randomly sampled RBG values, is measured through RMSE, MAD and MARD indexes introduced in Section 3.1. The mean value of each index obtained on the experiments of the test data set is then saved for the current MC iteration. Finally, after all the N MC iterations are performed, the sample distribution of the above indexes is obtained (see histogram in Figure 3), and compared with the result obtained with the specific calibration procedure under evaluation (red arrow in Figure 3). In particular, the percentage of the N MC iterations whose results are on the left of the red arrow (relative to the results obtained with the calibration scheme under test) represents a measure of the probability of reaching better accuracy calibrating in random time instants. In other words, if the red arrow is close to the distribution peak, the calibration scheme under test cannot be considered useful, since a random calibration strategy would give the same results.

## 4. Results

The Monte Carlo simulation is undertaken in order to assess how the indexes defined in the previous section are influenced by the number of the RBG points used for recalibration, namely, if the calibration rules proposed in the two case studies are really beneficial or if their improvements are only due to the consideration of more RBG points used for calibration.

### 4.1. Calibration Robustness against Sweat Events

Table 1 shows average and standard deviation (in parentheses) of RMSE, MAD and MARD obtained for the standard working case, *i.e.*, the calibration parameter *b* in Equation (2) is calculated only once, as baseline value, at the beginning of the experiment (first line in Table 1), and for the multiple calibration strategy under assessment, *i.e.*, *b* is updated using the first RBG available every time a sweat event is detected (second line in Table 1).

Both the test datasets are documented, *i.e.*, test dataset 2 when dataset 1 is used for model identification (1→2) and test dataset 1 when dataset 2 is used for model identification (2→1).

Statistical significance of the differences is verified analyzing the *p* values obtained by the application of the Student's t-test when both distributions under comparison result normal (a condition that is checked using the Kolmogorov-Smirnov test) and of the Wilcoxon Rank Sum test otherwise. In addition, since the considered accuracy indicators (RMSE, MAD or MARD) are calculated from data of independent experiments conducted in different study days, we assume that the values of both distributions under test are independent.

To assess if this improvement could be related to the higher number of RBG data points used, rather than to a real benefit deriving from recalibrating exactly after sweat events, the MC simulation described in Section 3.3 is performed.

**Figure 4.**
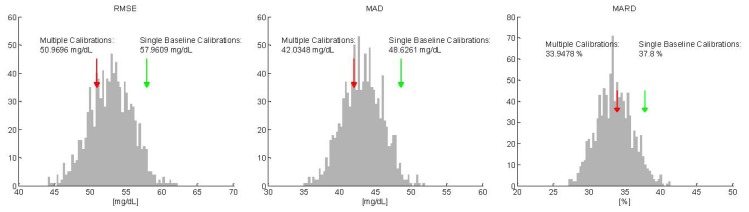
Monte Carlo simulations for case study #1 (sweat events). MC simulation results for RMSE (**left**), MAD (**middle**) and MARD (**right**) when database part2 is used as test sets. Distribution of MC model performance indicators (grey bars) compared with the accuracy results obtained with single initial baseline calibration (green arrows) and for the assessed multiple calibration strategy (red arrows).

For each of the 1,000 MC simulations, the mean accuracy of the random multiple calibrated glucose profiles was evaluated by the same key indicators used above. Then, the distributions of the key indicators on the 1,000 repetitions were compared with the mean values results in Table 1 and shown in Figure 4 for RMSE, MAD and MARD, respectively, only for one test data subsets (comparable results are obtained switching identification and test data sets—see 2→1 in Table 1). In Figure 4, the distribution of mean values of the key indexes calculated on the 1,000 MC simulations is depicted with grey bars, while the mean value obtained recalculating the calibration parameter after each sweat event is showed with a red arrow. Interestingly, the peaks of the distributions for the three indicators are exactly comparable with the results obtained with the proposed recalibration strategy (red arrows). In addition, we can note that a significant portion of the MC simulations produce a mean value lower than the one represented by the red arrow (24%, 31% and 54% for RMSE, MAD and MARD, respectively). Thus, the use of MC approach helps in understanding that the improvements (with respect to the single baseline calibration scenario) in terms of accuracy noticed in Table 1 are due to the increased number of RBG points used for calibration rather than to performing recalibration exactly after a sweat event to compensate for changes in the baseline of the main glucose signals induced by the event.

#### Remark

**Figure 5.**
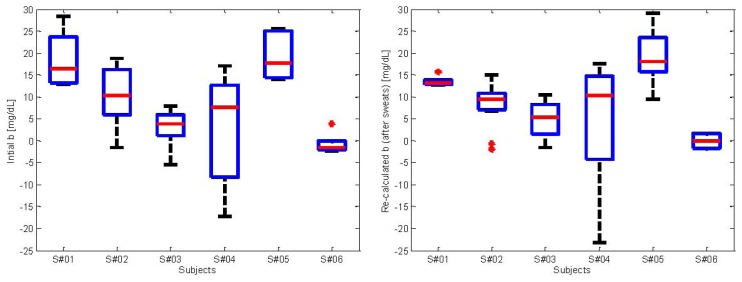
Range of *b* calculated at the beginning of each experiment for each of the six subjects (**left**) and after each detected sweat event (**right**).

In order to have some insight into the calibration parameter, in the left panel of Figure 5 we show the values of *b* calculated at the beginning of each experiment. In particular, each boxplot represents the distribution of the values of *b* calculated for each subject. The clustering per subject is clearly visible. Moreover, each subject presents values of *b* with different variability, as can be noticed from the boxplots presenting different width. The overall range of values assuming all the 6 subjects is approximately of ±25 mg/dL. This range highlights the importance of performing calibration, without which the error in estimating glucose would be much worse. The right panel of Figure 5 shows the re-calculated values of *b* after a sweat event is detected. The clustering per subject is still visible and resembles the distributions of *b* showed in the left panel. This further confirms that re-calculating *b* after sweat events is not necessary. Indeed, the values of *b* calculated initially are consistent with those after sweats.

### 4.2. Calibration Scheduling

**Figure 6.**
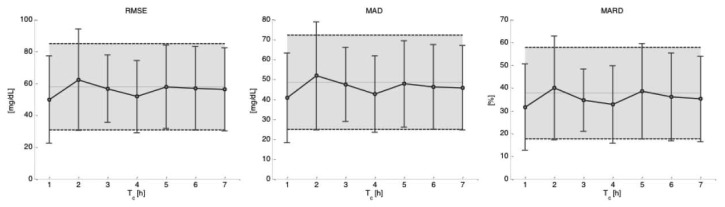
Effect of calibration scheduling on the accuracy of estimated glucose. Distributions (in terms of mean and standard deviation over the experimental sessions) of RMSE (**left**), MAD (**middle**) and MARD (**right**) for *T_c_* = 1, …, 7 h. The shaded area is only for comparison, and represents the results obtained only with the initial calibration.

Figure 6 shows the performances, in terms of mean and standard deviation over the experimental sessions of the test data subsets 2 (when data subset 1 is used for model identification), obtained for the different key indicators as a function of the different time intervals *T_c_* considered for recalculating b in Equation (2). The shaded area represents two standard deviations from mean results obtained when only the initial calibration is considered. As can be seen, there seems to be a reduction in the error measured by the accuracy indicators in performing re-calibration for *T_c_* = *1* and *T_c_* = *4 h*, although there is not a statistically significant difference with respect to the results obtained only with the initial calibration (see Table 2).

**Figure 7.**
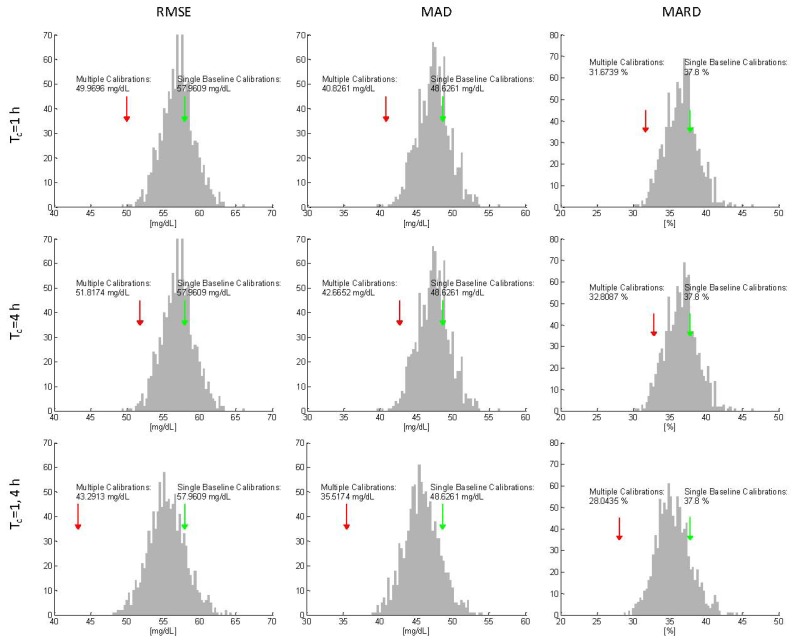
Monte Carlo simulations for case study #2 (calibration scheduling). MC simulation results for RMSE (**left**), MAD (**middle**) and MARD (**right**) for Tc = 1 h, Tc = 4 h and Tc = 1, 4 h. Distribution of MC model performance indicators (grey bars), for single initial baseline calibration (green arrows) and for the proposed calibration scheduling (red arrows).

Starting by taking as reference the mean values obtained recalculating *b* at *T_c_* = *1 h* [red arrow in Figure 7 (top)], we performed the MC simulation in order to test if a recalibration after 1h is really beneficial or only due to the consideration of one additional RBG point. In this scenario, for each of the N = 1,000 simulations, the recalibration is only one and its position in time randomly sampled over the interval 0 ÷ 7 h. Figure 7 (first row) shows, for the three proposed key indicators, the distribution of the results (grey bars) obtained with a recalculation of *b* in a random time instant. Differently from what observed in the scenario of Section 4.1, the mean values of the key indicators obtained with the MC simulation are all larger than those obtained with the scheduled *T_c_* = *1 h* (red arrow). In particular, only in 2% percent of the 1,000 MC iterations better results are achieved in terms of RMSE, suggesting that the increase in the observed accuracy (even if not statistically significant) is due to the time instant selected for calibration parameter update rather than to the random effect of adding a recalibration point. The same MC procedure was applied for testing the case *T_c_* = *4 h* and the results are similar to those found for *T_c_* = *1 h*, highlighting, again, the real advantage obtained applying the presented calibration scheduling (second row in Figure 7).

The results suggest that a calibration strategy involving three calculations of *b* (initial at time *t_i_* plus two after *T_c_* = *1* and *4 h*) could have the potential to further improve the accuracy of estimated glucose levels. Indeed, the results reported in the last row of Table 2, which are relative to performing recalibration both at *T_c_* = *1* and *4 h*, show a statistically significant improvement for the two of the three key indicators (RMSE with *p* = *0.005* and MAD with *p* = *0.04*). This important result is strengthened by the MC simulation (third row panels in Figure 6), that answers to the question if the same accuracy improvement can be obtained with random recalibration during the experiments. In fact, it is visible that the mean accuracy value obtained performing three calculations of *b* at *T_c_* = *0, 1, and 4 h* (red arrow) is lower than every single average value obtained performing the two additional recalibrations at random time instants (grey bar), confirming that additional calculation of the parameter vector *b*, whose scheduling is optimized over the time grid of the protocol, significantly improve the accuracy of the device and that this improvement is not due to having added two calibration points.

#### Remark

Although not shown here for the sake of space, comparable results are obtained if data subset 2 is used for model identification and data subset 1 for model test.

## 5. Conclusions

Tight monitoring of blood glucose levels is important to avoid the long- and short-term effects of diabetes complications. NI-CGM is appealing for obvious reasons related to patient comfort, although accuracy of current NI-CGM systems is not yet comparable to that of enzyme-based needle sensors which measure directly in the subcutis.

In this work we considered data from a recently proposed Multisensor device [17]. The modeling procedure for multi-sensor data relies on a calibration step, which exploits a RBG concentration value for calculating the baseline glucose provided by a fingerprick. A MC simulation technique was proposed to assess robustness of this calibration step in two situations.

In the first situation, the robustness of this specific calibration procedure against sweat events is assessed by comparing its accuracy with that achievable by repeating the calibration every time a sweat event occurs. Results showed that multiple calibrations performed after sweat events leads to a slight, but not statistically significant, improvement, consisting in a decrease of variability of the indexes widely used in the diabetes community to assess CGM sensors accuracy. The performed MC simulation demonstrated that this improvement can be attributed more to the exploitation of multiple RBG samples rather than to a benefit of updating the calibration parameter *b* after sweat events, highlighting that adjustments based on glucose fingerprick measurements every time a sweat event occurs are not necessary. From a pragmatic perspective, this conclusion is very important for obvious reasons related to patient's comfort, acceptance and to the proposed every-day usability of the device.

In the second case study, the MC methodology was then used to investigate if a calibration time scheduling could improve accuracy of estimated glucose profiles, and for confirming which is the time interval giving the highest performance improvement. Results showed that a calibration scheduling consisting in two recalibrations performed at *T_c_* = *1* and *4* hours, respectively, increases the accuracy of estimated glucose profiles, with a statistically significant improvement for RMSE and MAD. Thanks to MC simulation, we were able to demonstrate that this improvement is due to the proposed calibration scheduling and not to having added two recalibration points. This result is important, since it allows understanding that a multiple recalibration could be useful to compensate those effects that are not yet properly modeled or taken into account by the black-box multivariate linear regression model used to combine multisensory signals to derive an estimation of glucose concentration.

The work shows that improved point accuracy may be obtained through calibration strategies validated with a MC methodology. Nonetheless, accuracy of the considered Multisensor NI-CGM device is not at the same level of needle-based minimally invasive CGM devices (e.g., having MARD ranging from 11.8 to 20.2% [34]). However, as quantitatively assessed in [14], glucose trends exhibit a reasonably good precision and this can be potentially important in practice to complement information obtained by standard SMBG devices, for example obtaining in real-time a measure of the current short-term risk for the patient obtained by combining glucose level and trend according to the concept recently developed in [35].

It is useful noting that the proposed MC methodology for assessing the calibration step is not strictly related to the considered Solianis Multisensor, but has a more general domain of applicability; being NI-CGM multisensor data different from those presented in the paper not available to us, we can provide the reader with an only conceptual example of how the methodology could be used on a different device. The GlucoTrack, presented in [19], can be considered since it performs intermittent glucose monitoring with a different multi-sensor logic and a different calibration law with respect to the Solianis Multisensor. In particular, the GlucoTrack requires a specific calibration routine involving the parallel acquisition of six RBG values and GlucoTrack measures at pre-determined time instants for calibrating the device. The MC methodology can be used to verify the optimality of this calibration scheduling. Specifically, for each of the MC iterations, the GlucoTrack could be calibrated considering six random time instants where the pairs RBG and GlucoTrack values are available, obtaining (in the end) a distribution of the performance indicators (aggregating the results of the N MC iterations). This distribution can then be compared to the results obtained with the original calibration routine, verifying its optimality, following the procedure explained in Section 3.3. Finally, it is also worthwhile mentioning that the proposed MC methodology could be considered as a tool to optimize the choice of RBG samples to calibrate minimally-invasive CGM devices [36]. For example, the accuracy results obtained with a calibration model identified with one or more RBG samples at specific time instants can be compared with the distribution of the accuracy indicators obtained by the MC methodology.

Concluding, the presented methodology can represent a valid tool for driving the enhancement of the calibration step of NI-CGM devices, with the aim of further improving their usability in patients real-life.

## Figures and Tables

**Table 1.** Key indicator results for the single and multiple glucose calibration. Average and standard deviation (in parenthesis)—over experimental sessions—of RMSE, MAD, MARD obtained when database part 1 and database part 2 are used for model identification and model test, respectively, (1→2), or viceversa (2→1). Single Baseline Calibration: parameter b in Equation (2) is calculated only at the beginning of the experimental session; Multiple Calibrations: b in Equation (2) is updated every time a sweat event is detected. The p value indicates the statistical difference between the two calibration strategies according to the Student t-test.

	RMSE [mg/dL]		MAD [mg/dL]		MARD [%]	

	1→2	2→1	1→2	2→1	1→2	2→1

	*p* = 0.3	*p* = 0.7	*p* = 0.3	*p* = 0.6	*p* = 0.5	*p* = 0.7

Single Baseline Calibration	57.9 (27.1)	57.5 (25.1)	48.6 (23.7)	47.2 (21.8)	37.8 (20)	39.4 (20.1)
Multiple Calibrations (after each sweat event)	50.9 (20.8)	52.8 (19.5)	42 (19.8)	42.2 (15.5)	33.9 (18.8)	34.4 (10.9)

**Table 2.** Key indicators results for the calibration scheduling. Average and standard deviation (in parentheses)—over experimental sessions—of RMSE, MAD, MARD obtained when database part 1 and database part 2 are used for model identification and model test, respectively, along with the p values from the Student t-test for assessing the statistical differences between the key indicators between the initial baseline calibration and the proposed ones.

	**RMSE [mg/dL]**		**MAD [mg/dL]**		**MARD [%]**	

**Initial Baseline Calibration**	**57.9**		**48.6**		**37.8**	
	**(27.1)**		**(23.7)**		**(20)**	
Multiple Calibrations (after 1 h from initial)	49.9 (27.4)	*p* = 0.23	40. 8 (22.5)	*p* = 0.29	31.6 (18.9)	*p* = 0.34
Multiple Calibrations (after 4 h from initial)	51.8 (22.6)	*p* = 0.15	42.6 (19.1)	*p* = 0.17	32.8 (17)	*p* = 0.17
Multiple Calibrations (after 1 and 4 h from initial)	43.2 (21.8)	*p* = 0.005	35.5 (17.9)	*p* = 0.04	28 (15)	*p* = 0.1
